# The “A to Z” of Managing Type 2 Diabetes in Culturally Diverse Populations

**DOI:** 10.3389/fendo.2018.00479

**Published:** 2018-08-28

**Authors:** A. Enrique Caballero

**Affiliations:** Office for External Education, Harvard Medical School, Boston, MA, United States

**Keywords:** type 2 diabetes, race, ethnicity, culture, health care disparities, social medicine, treatment, vulnerable populations

## Abstract

Type 2 diabetes affects racial/ethnic minorities at an alarming rate in the US and in many countries around the world. The quality of health care provided to these groups is often suboptimal, resulting in worse patient-related outcomes when compared to those in mainstream populations. Understanding the complex biological elements that influence the development and course of the disease in high-risk populations is extremely important but often insufficient to implement effective prevention and treatment plans. Multiple factors must be addressed in routine diabetes clinical care. This paper discusses various key factors, organized in alphabetical order. These are acculturation, biology, clinician's cultural awareness, depression and diabetes-specific emotional distress, educational level, fears, group integration, health literacy, intimacy and sexual dysfunction, judging, knowledge of the disease, language, medication adherence, nutritional preferences, other forms of medicine (alternative), perception of body image, quality of life, religion and faith, socio-economic status, technology, unconscious bias, vulnerable groups, asking why?, exercise, “you are in charge” and zip it! Considering these factors in the development of type 2 diabetes prevention and treatment programs will help improve diabetes-related outcomes in culturally diverse populations and reduce health care disparities.

## Introduction

Modern societies around the world are integrated by culturally diverse populations and thus health care professionals frequently face the challenge of providing care to a wide variety of patients. The management of diabetes and other chronic diseases can be particularly difficult since multiple biological, social, psychological and cultural factors play a role in the development and progression of these conditions. Health care providers often have limited awareness of how these factors must be addressed in clinical practice, particularly in our current environment when very limited time to interact with patients is available during clinical encounters. In addition, few health care settings have truly embraced the development and implementation of effective culturally oriented programs that make people from different populations feel they are truly heard and understood.

It is somewhat puzzling that most patients with diabetes have not achieved treatment goals at a time in history when we are enjoying such vast scientific knowledge about the disease, coupled with the availability of effective therapeutic strategies to alter its course (1). Perhaps we have focused too much on the medical/biological aspects of the disease and we have not fully addressed educational, social, financial, psychological and cultural determinants of health and disease when designing prevention and treatment programs for our patients.

In this work, I first share some basic concepts about race and ethnicity as well as demographic data in the United States of America (US) as a true example of a multicultural society. I then discuss some of the main factors that influence diabetes care so that readers are aware of their relevance and how to address them in clinical practice. I have organized these factors in alphabetical order to create an “A to Z” list. There is no particular order of importance—I believe they all are important!

## Race and ethnicity

*Race* is primarily based on our phenotype and determined by shared genetically transmitted physical characteristics. On the other hand, *Ethnicity* is based on our cultural, linguistic, religious and national background. It relates to our family life, beliefs, rituals and even food preferences (2).

There may be some confusion with the use of these terms. For example, *Latino* or *Hispanic* is not a race, it is at ethnicity. In the US, it alludes to people from Latin America (and Spain) who share a specific cultural and linguistic background along with certain traditions and social characteristics. At the same time, from the racial perspective, *Latinos/Hispanics* have three main genetic backgrounds: white, African-American, and/or Native Indian. These backgrounds are often mixed, creating a very heterogeneous population (3).

## Culturally diverse populations in the United States

The main racial/ethnic minorities in the US are Latinos/Hispanics, African-Americans, American Indians, Alaskan natives, Asian and Pacific Islanders, Southeast Asians, and Arabs. These groups have generally grown at a faster pace than the non-Hispanic white population. US Census Bureau (4). At the present time, these groups combined represent approximately 35% of the total population. It is predicted that this figure will increase to 50% by the year 2050. US Census Bureau (4). The US and many other countries around the world are true mosaics of multiple cultures. Current and projected percentage of the US population by race and ethnicity from the year 2000 to the year 2050 is shown in Table 1 (4).

**Table 1:** Current and projected percentage of the US population by race and ethnicity from the year 2000 to 2050.

**Population or percent and race or Hispanic origin**	**2000**	**2010**	**2020**	**2030**	**2040**	**2050**
White alone	81.0	79.3	77.6	75.8	73.9	72.1
Black alone	12.7	13.1	13.5	13.9	14.3	14.6
Asian alone	3.8	4.6	5.4	6.2	7.1	8.0
All other races	2.5	3.0	3.5	4.1	4.7	5.3
Total	100	100	100	100	100	100
Hispanic (of any race)	12.6	15.5	17.8	20.1	22.3	24.4
White alone, not Hispanic	69.4	65.1	61.3	57.5	53.7	50.1

*Rows 1–4 represent main races and add to 100%. Rows 6 and 7 refer to the presence or absence of Hispanic ethnicity and are different categories from race*.

## Type 2 diabetes in racial/ethnic minorities

**Figure 1:**
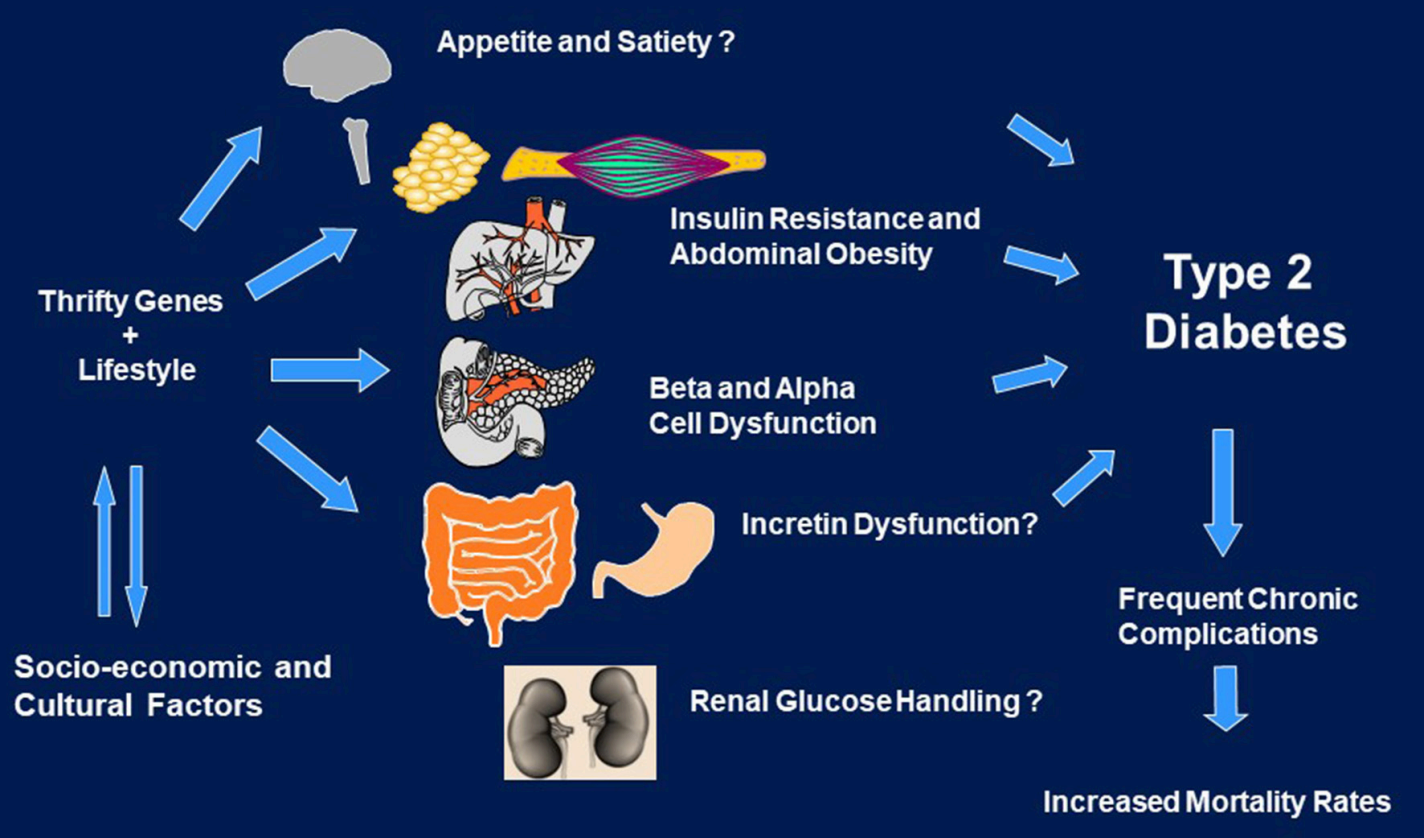
Genes, environment, and social/cultural factors in the development and course of diabetes in culturally diverse populations.

Racial/ethnic minorities usually have higher prevalence rates of type 2 diabetes and many of its complications in comparison to mainstream groups (5–9). Type 2 diabetes is a heterogeneous disease that results from the combination of genetic predisposition and environmental factors (Figure 1). The concept of a “thrifty gene” that allowed some indigenous groups who were exposed to alternating periods of feast and famine to develop the ability to efficiently store fat during periods of plenty to better survive famine has been intriguing (10). If certain, it could provide the basis for a mechanism that once protective has now become deleterious since food supplies are more regular and abundant among some of these groups. Whereas no single gee has been identified to explain such high tendency to diabetes in these populations, the field of genetics research has rapidly advanced and has provided insight into multiple abnormalities that play a role in subgroups of patients (11). In addition, lifestyle factors such as inappropriate eating habits coupled with reduced physical activity are well known to influence the development of type 2 diabetes, both of which are frequently found in culturally diverse groups at high risk for the disease (6, 9). Finally, social and cultural factors also influence the development and progression of the disease (9) (Figure 1).

## Health care disparities

Racial/ethnic minorities usually receive a lower quality of health care in comparison to mainstream groups. The Institute of Medicine in the US reported the presence of significant health care disparities between racial/ethnic minorities and the non-Hispanic white population after accounting for factors such as health care system access, age, socioeconomic status, stage of presentation and existing comorbidities (12). These disparities were identified in public, private, managed care, teaching/academic and community health centers (12). Health Care disparities represent a complex and challenging situation in which health care provider, patient and health care system's characteristics are deeply intertwined. Understanding and addressing each of these elements is crucial to advance the work in eliminating health care disparities in the US and around the world. Diabetes prevention and treatment programs must incorporate a myriad of strategies based on patients' social and cultural characteristics and the communities where they live. In addition, efforts to improve health care providers' cultural and social awareness are highly needed. Ultimately, health care systems must embrace and support the development of culturally and socially oriented clinical settings and programs to improve the lives of people from underserved populations.

## The A to Z list for managing type 2 diabetes in culturally diverse populations (Figure 2)

### Acculturation

*Culture* alludes to beliefs, behavior patterns, and all other products of human thought and work in a certain community (2). *Acculturation* refers to the adoption of specific elements of a group's culture by an individual not originally from that group (2). For immigrants to the US and other developed countries, it pertains to the incorporation of mainstream cultural aspects into regular beliefs and behavior patterns. The influence of acculturation on health care behaviors has been studied for a long time without uniform findings. Low acculturation has been related to worse health care behaviors, partly due to lower education levels and access to health care (13–15). On the other hand, low acculturation manifested by the preservation of own traditional diets by immigrant groups has been shown to be beneficial (16). Along this line, high acculturation levels are associated with high rates of type 2 diabetes, likely related to the adoption of carbohydrate- and fat-rich foods as well as low levels of physical activity (13–15). Recent data of Chinese immigrants to Australia expand on the influence of acculturation on the increased prevalence of cardiovascular disease (CVD) risk factors (17). However, a high acculturation level can also lead to an improvement in dietary habits and physical activity, likely related to increased education level and socio-economic status (18).

**Figure 2:**
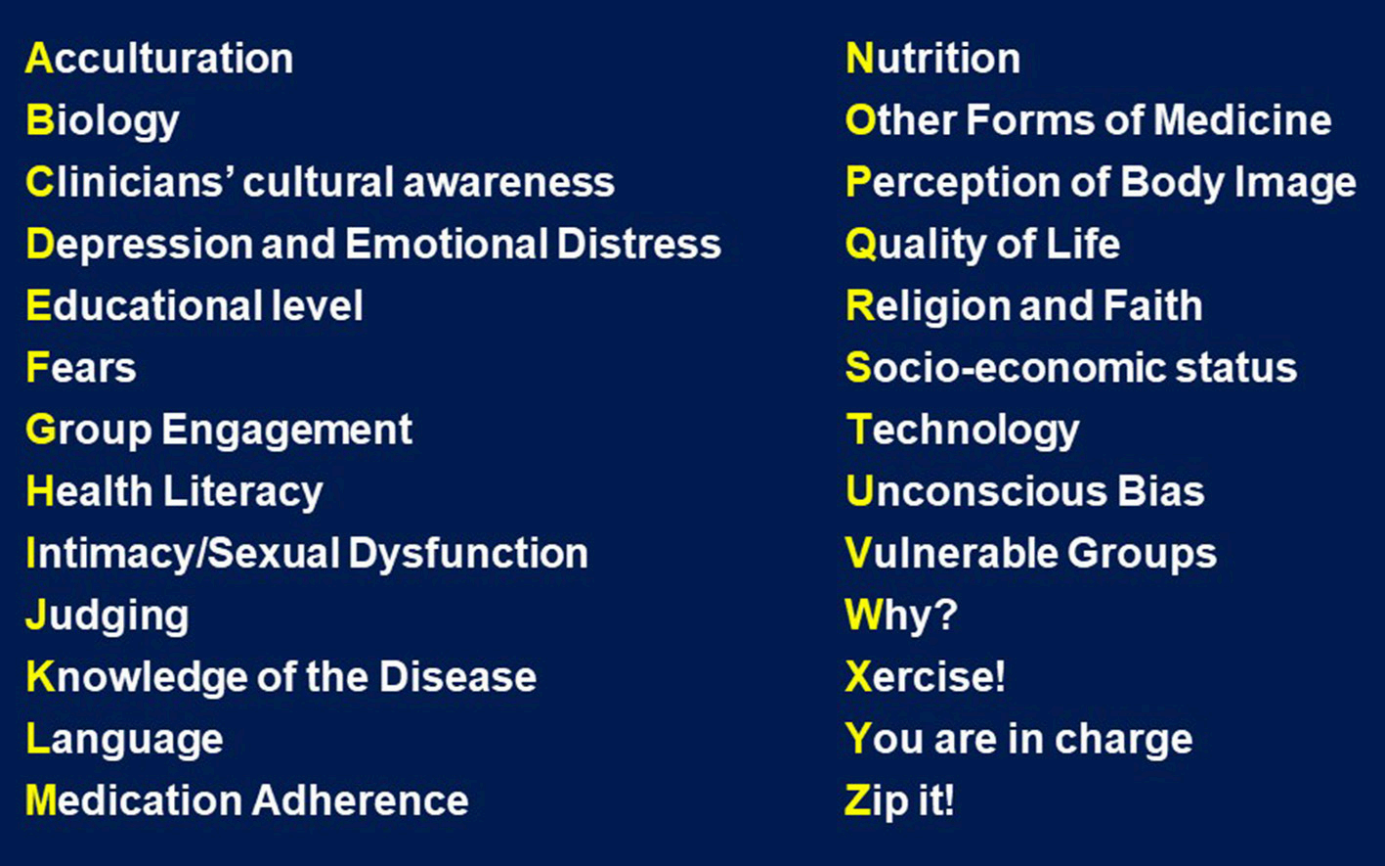
Factors to consider when managing culturally diverse patients with type 2 diabetes.

Evaluating acculturation level in clinical practice is not easy. Many reports consider language preference as a good estimate of the degree of acculturation (18). However, no uniform instrument to assess acculturation exists as it would need to be adapted to the population being studied and to the goals of the evaluation (19). Health care providers should openly ask patients about lifestyle patterns they have adopted from mainstream culture that may influence diabetes-related self-care behaviors.

### Biology

The pathophysiology of type 2 diabetes involves abnormalities at multiple organ and system levels. Pancreatic beta and alpha cells, the adipose tissue, the skeletal muscle, the liver, the kidney and the gastrointestinal and central nervous systems participate in the development and progression of the disease (20). Differences in some of these pathophysiological defects among racial/ethnic groups have been reported but scarce information exists about other abnormalities. A detailed review of these differences is beyond the scope of this manuscript. However, some interesting findings can be highlighted.

Insulin sensitivity has been shown to be reduced in many groups at high risk for type 2 diabetes. For instance, healthy normoglycemic adult Mexican-Americans, African-Americans, and Asian-Americans exhibit lower insulin sensitivity in comparison to white counterparts of similar body weight (21). These differences have also been shown in Hispanic-American and African-American youngsters after adjusting for differences in body fat (22). Most US racial/ethnic minorities such as Latinos/Hispanics, African-Americans, Asian-Americans, South East Asians, American Indian, Alaska Natives and Arab-Americans usually have higher rates of insulin resistance than the non-Hispanic white population (23). Most likely, increased insulin resistance is related to genetic and lifestyle factors (20).

Compensatory insulin production to insulin resistance seems to also be affected in some of these groups (24–28). Some studies suggest that beta cell function tends to deteriorate frequently and at a fast pace in high-risk populations contributing to the genesis of type 2 diabetes (24–28). The precise mechanisms involved in beta cell dysfunction in these groups have not been fully identified.

Body fat accumulation increases the risk for type 2 diabetes and CVD (20). Obesity rates are significantly higher among some racial/ethnic minorities in comparison to non-Hispanic white counterparts (23, 29). For example, 80% of non-Hispanic black women were considered overweight in a recent report (29). Overall, the rates for obesity and the cardiometabolic syndrome are extremely high in minority populations leading to alarming morbidity and mortality rates (30). Multiple genetic, lifestyle, social and cultural factors likely contribute to the high obesity rates among these groups.

The amount, type and location of body fat contribute to the risk of type 2 diabetes and related disorders (20). Increased content of visceral fat usually leads to decreased insulin sensitivity and impaired endothelial function (31). Interestingly, whereas overall obesity rates are usually higher in some racial/ethnic minorities in comparison to whites, African-Americans have been identified with lower visceral fat content than non-Hispanic whites with similar body mass index (BMI) (32). In contrast, South East Asians usually have lower rates of obesity but higher visceral fat content when compared to Caucasians of similar BMI (33). Therefore, both BMI and waist circumference (a proxy for visceral fat) are useful screening tools that help to identify high-risk individuals. The measurement of waist circumference in clinical practice should be routinely performed to assess abdominal obesity^1^. Be mindful that different cutoff levels to identify abdominal obesity have been established for some populations^1^. Particular attention must be paid to Asian patients who may not be considered overweight or obese according to traditional standards but who tend to accumulate high levels of intra-abdominal or visceral fat leading to increased insulin resistance (34).

The problem of overweight and obesity is alarmingly increasing in racial/ethnic minorities, affecting many young individuals (29). We have found that normoglycemic, normotensive overweight Hispanic children, adolescents and young adults exhibit insulin resistance and endothelial dysfunction, suggesting a clear path to type 2 diabetes and CVD at early ages (35, 36).

We still need more research that aims at evaluating biological differences among racial/ethnic groups to better understand similarities and differences in all components of the pathophysiology of the disease. Understanding this phenomenon will ultimately help us design more effective therapies for specific patient populations.

### Clinician's cultural awareness

Are we fully aware of our patients' culturally driven beliefs and behaviors? If so, do we appreciate them and respect them? We all have our own culture. Culture influences our thoughts, beliefs, and behaviors. Respecting cultural differences is essential for a meaningful and productive interaction with each other. Cultural competence (awareness) alludes to health care providers' knowledge and skills to understand, respect, appreciate and interact with patients from cultures other than their own (37).

Establishing a good patient-provider communication is key to help patients improve diabetes self-care behaviors (38). Cultural awareness can positively influence the relationship with patients. Being culturally aware does not strictly mean speaking the same language that patients do. In fact, although being able to interact with patients in the same language is extremely helpful, it is not often necessary. A great communication can be established with patients through the help of a qualified interpreter. Cultural awareness is really more about becoming interested in patients' health beliefs, habits and explanatory models of health and disease and being able to interact with them in a genuine and respectful manner.

The need to improve our cultural awareness as health care providers is now widely recognized (39, 40). Continuing medical education courses are starting to regularly include activities that aim at improving physicians' knowledge and skills to address social and cultural aspects in health care. Similarly, medical schools are integrating cross-cultural health care models and strategies in their curriculum. All these efforts are likely to contribute to improve patient related outcomes and reduce health care disparities (41).

### Depression and diabetes-specific emotional distress

The prevalence of depression among patients with diabetes is two to three times higher than in the general population (42). The diabetes—depression relation is usually bidirectional, with diabetes increasing the likelihood of depression and depression contributing to type 2 diabetes, usually through the presence of a lifestyle leading to obesity as well as through other mechanisms (42). It is believed that dysregulation of the hypothalamic pituitary-adrenal axis and of neurotransmitter systems, especially the monoaminergic system, oxidative stress and neuroinflammation may influence this relationship (42). Depression often predicts poor diabetes-related health outcomes (43).

Depression is common in racial/ethnic minorities and influences multiple health care behaviors (44). Multiple psycho-social factors are known to impact diabetes-related self-care behaviors in these groups, including low socioeconomic status, low acculturation, and sense of isolation (45). For many immigrants, adapting to a new social and cultural environment is often challenging and stressful. Some may never feel fully integrated into mainstream society. Elderly Puerto Ricans living in the East coast of the US have been reported to have higher rates of cognitive impairment, depression and some chronic conditions like diabetes than non-Hispanic whites living in the same geographical area (46).

Unfortunately, patients' depression is not frequently identified in routine clinical care (47, 48). Multiple instruments have been developed to identify depression in research and in clinical practice (49). The routine implementation of these strategies in busy practices may be challenging but efforts to identify practical and sustainable efforts to detect depression in patients with type 2 diabetes is highly recommended. Sometimes, just asking simple questions such as “Have you lost interest in your day-to-day activities lately?” or “Do you often feel sad?” may be a good way to start a conversation in this area. A careful and thorough evaluation and proper referral to a specialized team may be required.

Diabetes-related emotional distress is also a common condition among patients with type 2 diabetes. It relates to the psychological impact of having diabetes and the need to follow multiple recommendations every single day (50). The routine evaluation of diabetes-related emotional distress is highly recommended (51). Readers should become familiar with available instruments to identify it in clinical practice (51). Comprehensive culturally oriented programs that address depression and emotional distress can improve diabetes-related outcomes (52).

### Educational level

Diabetes management and prevention programs require patients to engage in multiple complex self-care behaviors that are sometimes difficult to communicate and comprehend. Not surprisingly, the prevalence of type 2 diabetes and CVD has been closely linked to participants' educational background. For example, a study in Washington found that Japanese-American men with high-school or higher education level had a lower frequency of these diseases compared to those with a technical school education level (53). In the analysis of contributing factors, it was found that those with lower educational level had higher BMI and worse lipid profile than those with higher educational level. Multiple socioeconomic, lifestyle and cultural factors likely explain the association between educational attainment and the prevalence of type 2 diabetes and CVD.

Educational level has also been correlated with diabetes control and diabetes-related outcomes (53, 54). A recent international clinical trial found that patients with type 2 diabetes and low educational level had an increased risk of vascular events and death in comparison to those with high educational attainment after controlling for CVD risk factors (55).

Educational and socioeconomic levels are closely linked. Self-care behaviors such as following a healthy meal plan and engaging in regular physical activity are influenced by both educational and socioeconomic levels. As a result, increased body weight has been found to have an inverse correlation with socioeconomic and educational levels (56).

Interestingly, not all studies have found a strong correlation between patient's educational background and lifestyle changes in type 2 diabetes (57). This association can be influenced by many patient related factors. Low educational level is not considered a direct cause of diabetes and/or worse diabetes related outcomes but rather an important factor deeply intertwined with multiple health care behaviors that influence the development and progression of the disease. Clinicians should take patients' educational background into consideration when designing any individual or group education program or clinical intervention to maximize their benefits.

### Fears

Fears are common in patients with type 2 diabetes and are usually interwoven with values and health beliefs. Some may have emerged from personal experiences or from collective myths frequently embedded in the corresponding culture. It is understandable that many patients fear the development of the disease and its potentially devastating complications. However, some fears may affect patients' acceptance of the disease and adherence to a diabetes treatment plan (58). A good example of a fear that negatively affects patients' adherence to a treatment plan is that based on the misconception that a medication causes an unproven harm. Among Latinos/Hispanics and in other populations, the fear of starting insulin therapy has been deeply embedded in their culture for many years. Insulin is often considered a death sentence. Patients consider that insulin initiation will invariably lead to the appearance of severe and fatal complications (59). There is simply a chronological association between starting insulin therapy and the identification of some diabetes related chronic complications. However, patients have long blamed insulin for the development of cardiovascular disease, nephropathy, eye disease and other complications (59). Among Latinos/Hispanics, they myth of insulin causing blindness is particularly common. Therefore, a tremendous fear and hesitation to start insulin therapy is often identified (59, 60).

Clinicians should ask patients with diabetes if they have any fear related to any glucose lowering medication or any other therapy that may be introduced. Openly asking patients about any fear to start insulin treatment is often an appropriate first step (61–63). Educational activities that aim at dispelling myths and fears are highly recommended. Understanding patients' health belief systems can help develop congruent intervention programs based on individual and group characteristics.

### Group engagement

We are gregarious by nature. We like to belong to groups of people. The closest group for most people is the immediate family. Interestingly, populations differ in the level of closeness and dependence among family members. Among Latinos/Hispanics, Arabs, Asian Indians and other groups family needs are often more important than those of any individual (23). Therefore, engaging relatives in clinical and education activities may allow patients to build a positive support system. Patient's close friends may also contribute to this network. Some reports suggest that family structural togetherness improves quality of life and satisfaction among patients with diabetes (64–67). Family support may in fact contribute to improve diabetes control (68). However, it is not completely clear what behaviors in families truly impact diabetes-related outcomes (64).

In addition, group activities may be beneficial in both clinical care and patient education settings. Group classes have been long recognized as a strategy to empower patients through peer support (69). More recently, shared medical appointments or group medical visits have emerged as an effective strategy to provide clinical care to patients (70). However, more research is needed to identify best candidates for this type of clinical care model. It is important to remind ourselves that in some cases, patients may truly prefer individual rather than group activities and therefore, different types of programs may need to be developed.

### Health literacy

Health literacy refers to patient's ability to obtain, process, and understand basic health information and services to make appropriate health decisions (71). Educational level and health literacy are not the same. In fact, someone may have a high educational level but low health literacy if that person has not been frequently and adequately exposed to the health care field.

Patients with type 1 and type 2 diabetes frequently have limited health literacy and has been linked to inadequate diabetes self-care management behaviors (71). In addition, low health literacy has been found to be associated with worse diabetes-related outcomes in some but not all studies (72–74). Health literacy is considered an important contributing factor to diabetes related self-car behaviors among Latinos, African-Americans, Asian/Pacific Islanders, and white Americans (75).

Due to these findings, diabetes self-management education should be tailored to specific populations, taking into consideration health literacy levels as well as type of diabetes, racial/ethnic, social, cognitive, educational and cultural factors (76). Involving patients in the development of own patient education programs and materials is recommended. This strategy may be particularly useful in those with low health literacy levels (77). Health literacy can be assessed in different ways. The Health Literacy Scale is a frequently used instrument. However, the structural validity of this scale needs to be further established, particularly in languages other than English (78).

Numeracy, the ability to understand and work with numbers has also received increased attention in the field of diabetes (78). We often ask patients with diabetes to use numbers when taking medications, adjust dosages, following scales, etc.

It is recommended to identify population specific instruments that accurately assess patients' health literacy and numeracy and utilize them as routine tools in clinical practice. Considering them for the development of targeted diabetes management programs may contribute to improving clinical outcomes in patients with type 2 diabetes from culturally diverse populations.

### Intimacy and sexual dysfunction

Sexual dysfunction occurs frequently in men and women with type 2 diabetes. In men, erectile dysfunction, ejaculatory dysfunction, and loss of libido are the most common disorders. In women, decreased libido and painful intercourse may occur. It is estimated that approximately two thirds of men with type 2 diabetes have erectile dysfunction, a much higher figure than in the general population (79). The mechanisms for sexual dysfunction involve microvascular and nerve damage. As in the general population, psychological factors are often involved in the development of these disturbances. Sexual Dysfunction can also negatively impact patients' psychological status.

Erectile dysfunction, considered a microvascular abnormality in patients with diabetes is closely related to CVD. Patients with diabetes and erectile dysfunction should undertake a thorough clinical evaluation to identify occult CVD (80).

Addressing intimacy and sexual dysfunction is important in diabetes care. The routine identification and evaluation of sexual problems by the clinical care team should be considered (81, 82). Discussing issues related to intimacy and sexual dysfunction with patients from culturally diverse populations requires health care providers to familiarize themselves with appropriate ways to initiate and engage in these conversations. Culture definitely influences patient's approach to these matters.

Interestingly, when properly addressed, both men and women with type 2 diabetes may adequately engage in diabetes related self-care behaviors to improve sexual performance.

### Judging

In our quest to guide patients with diabetes on how to improve their self-care behaviors, we may erroneously become the toughest judges of their performance. A judgmental, dictatorial and unilateral approach in provider-patient communication is unlikely to provide optimal results. The opposite of being judgmental is to be uncritical. This does not mean not to have an opinion about patients' performance but to express it in a respectful and positive way. A respectful approach to patients' behavior, preferences and culture is the first step to engage patients in a true collaborative model to make better decisions in health care.

Shared decision making is recommended in the management of type 2 diabetes. It is inherent to a patient-centered approach that has demonstrated to improve diabetes care (83). It focuses on engaging the patient in their own health care. To achieve this, eliciting and sharing accurate information about their health is crucial. Several patient engagement strategies have been explored to engage and motivate patients in routine clinical encounters. Motivational interviewing is one of these strategies. It allows health care professionals to demonstrate empathy, deal with patient's resistance to particular recommendations, discuss differences in opinion and ultimately support self-care behaviors (84).

In culturally diverse populations, effectively engaging patients in their own care requires the development of linguistic and culturally oriented programs and clinical settings. We need less judging and more understanding behaviors. Instead of blaming patients for not achieving treatment goals, perhaps we should routinely ask ourselves: “What can I do differently to help my patient?”

### Knowledge of the disease

Patients' diabetes knowledge is associated with diabetes self-care behaviors (85). It is considered that improving self-management behaviors is likely to lead to better diabetes control and, hence, a lower risk of diabetes-related complications. From that perspective, improving patient's diabetes knowledge is an important component of the standards for diabetes self-management education and support (85). Improving patients' diabetes knowledge must be done in a culturally and linguistically oriented fashion (86, 87). Identifying diabetes knowledge gaps and developing targeted education programs for culturally diverse populations is highly recommended. Involving patients in the creation of these programs may contribute to enhance the impact of these interventions (88, 89). The educational curriculum should be based on recommended self-care behaviors and patients' characteristics, knowledge gaps, goals and their cultural and social context (85–90).

When planning patient education programs to improve knowledge and self-care behaviors in culturally diverse populations, the inclusion of peers and community health leaders/workers may lead to improved patient-related outcomes (91–95).

### Language

Language barriers are often encountered in diabetes care. Health care providers and patients can't often interact in the same language. As a result, patients are sometimes not able to directly and fully express their thoughts, feelings and concerns. Language barriers are an obvious limitation in clinical encounters and have been shown to negatively impact clinical outcomes (96, 97).

Some patients prefer to interact with clinicians who share their cultural and linguistic background. This strategy has shown a beneficial impact on adherence to therapy and maintenance of appointments (98). However, it is very difficult to match patients with health care providers that speak the same language. There are simply not enough physicians who can take care of the number of people from culturally diverse populations in the US and in other multicultural societies (4, 96, 98). It is extremely important to expand and foster the training of clinicians that truly reflect the patient population in the community and the health care system.

Adequately trained interpreters are necessary and are a great addition to the health care team (99–102). Sometimes, patients' family members are asked to serve as interpreters. Whereas they may be able to relate valuable additional information about the patient to the health care provider, they may not be objective enough to translate all information without any bias and may focus on what he/she believes is important for the patient to hear and/or express.

Clinicians and their teams should anticipate patient's language needs. If possible, patients must be offered the possibility of interacting with clinicians who have the same cultural and linguistic background. However, efficient interactions are expected with the use of well trained and skilled interpreters if necessary. In the end, what matters most is that health care professionals genuinely become interested in establishing respectful, engaging and efficient interactions with all patients, regardless of race, ethnicity, culture and language.

### Medication adherence

The prevalence of adherence to diabetes medications ranges from 38.5 to 93.1%, depending on how it is evaluated and the population in which it is studied. On average, only 20% of studies in the field report medication adherence rates higher than 80% (103).

Decreased adherence to glucose lowering medications contributes to the progression of type 2 diabetes and its complications (104). It is estimated that for each 10% increase in the diabetes medication possession ratio, the mean glycated hemoglobin (HbA1c) decreases by 0.24% (104). Consistent with these findings, adequate medication adherence is associated with improved diabetes-related quality of life, decreased risk of hospitalization and reduced all-cause mortality in patients with type 2 diabetes (104, 105).

Multiple factors influence adherence to diabetes medications. The presence of depression and the cost of medications are among the most common predictors of adherence to therapy (105). Factors such as age, race, health beliefs, medication cost, co-pays, insulin use, health literacy, primary non-adherence, and early non-persistence affect adherence to medications as well (105). In addition, adherence is impacted by decreased tolerance and the need to take medications several times a day (106). Forgetfulness, side effects and polypharmacy may also play a role in decreased adherence to medications (107).

It is recommended that clinicians routinely address adherence to medications. While improved adherence would likely increase medication costs, it would decrease overall health care resource utilization and costs (104, 108).

Adherence to medications has been reported as a more significant problem among racial/ethnic minorities (109). Strategies that include a multi-disciplinary approach, integrative health coaching, case managers and community-based activities to improve adherence to medications should be considered in routine diabetes care. The cost of medications should always be considered when making decisions on what treatment strategy to implement in patients with type 2 diabetes (110, 111).

### Nutritional preferences

Medical nutritional management is an important component of diabetes care. Addressing nutritional preferences in patients with type 2 diabetes form culturally diverse populations is one of the most challenging tasks for any health care provider. Each racial/ethnic group has unique and preferred foods. Nutritional habits may differ not only by country of origin but sometimes even by city within any given country. In addition, the words that are used to describe same foods across countries may be totally different. For example, in Latin American countries, Mexicans may refer to a banana as “platano”, Venezuelans as “cambur” and Puerto Ricans as “guineo” (3). This is one of the reasons why patient education materials in Spanish don't usually appeal to all Latinos/Hispanics in the US. They often need to be tailored to each subgroup. Therefore, there is no “Latino diet” or “Asian diet” as preferences and food-related language may vary among population subgroups.

Proper culturally oriented nutritional evaluation must take place with all patients with type 2 diabetes. Dieticians and other nutrition experts who are familiar with patients' cultural and linguistic background can be very helpful in this process. Foods that patients from culturally diverse populations prefer and avoid must be identified to develop appropriate interventions (112). Patient education programs must include activities to improve nutritional preferences and should be tailored to patients' cultural and social background. In addition, health care professionals must identify local and community resources to help patients improve their food choices. Resources from regional and national academic organizations can be very helpful as well.

Even though recommendations on how to improve food choices are routinely given to patients, the implementation of these changes is not always possible. Food insecurity is an increasing problem in the US and other countries. It relates to the unreliable availability of nutritious food and the inability to consistently obtain food in certain communities (113). In the US, it is estimated that around 14% of people are food insecure. This figure is higher in some racial/ethnic minority groups such as Latinos/Hispanics and African Americans (113). This is a major social problem. However, health care providers must be aware of the nature and magnitude of the problem and when possible provide counseling to patients about how to overcome some barriers to access recommended foods. In addition, guiding patients on how to improve food purchasing behaviors can be highly beneficial in patients with type 2 diabetes (114).

### Other types of medicines (alternative)

At least half of patients with type 2 diabetes use some form of alternative medicine method (115). The most frequent are herbs and plants, homeopathy, naturopathy, yoga, relaxation, massage, acupuncture, chiropractic care, ayurveda, hypnosis, energy healing, Reiki therapy, chelation and biofeedback. In addition, dietary supplements are frequently utilized by patients with type 2 diabetes (116).

Alternative medicine use may lead to improved engagement with the health care system. Patients who follow some of these methods have been found to receive more frequent preventive care and regularly attend appointments with their primary care providers (115). Patients who chose to use an alternative medicine method may develop a more proactive approach to their own diabetes care. It is also possible that use of routine health care services may trigger the use of alternative medicine methods (117).

The impact of alternative medicine methods on diabetes related outcomes is still uncertain. Some studies are starting to emerge in this field. For example, yoga showed a beneficial effect on diabetes and dyslipidemia control in a group of patients with type 2 diabetes (118). It is encouraging to see that formal clinical trials to evaluate the impact of commonly used alternative medicine methods are being developed.

Clinicians should routinely ask patients if they use any alternative medicine method. In general, patients don't need to be dissuaded from continuing with their therapies as long as they follow recommended diabetes self-care behaviors. However, it is important to get familiar with the methods patients follow and their potential impact on diabetes care. Respectful discussions about their use should take place.

### Perception of body image

We perceive our body image in a unique way. Our perception is modeled by individual and cultural factors. In many groups, being overweight is a sign of prosperity as it reflects access to adequate amounts of food and a “good life.” Thus, increased body weight or robustness usually equates being healthy. This may be particularly common among populations who migrate to industrialized countries. Gaining a few pounds or kilograms goes along well with and improved financial and social status.

However, our culturally driven perception of body weight may start early in life. In many societies, children are encouraged to finish all the food on their plates regardless of their hunger level. We congratulate them and reward them when they do so. Unfortunately, we teach our youngsters to eat more than what they really need.

In some studies, African-American and Latino/Hispanic women with type 2 diabetes have expressed that being overweight reflects a healthier status than having a normal weight (119, 120). These findings should not suggest that patients in these groups aim at being overweight or obese. Most of them would like to lose weight. However, their weight loss goals may be very different from our recommended targets. Therefore, the discussion of body weight should consider patient's individual perception of body image (3, 23).

### Quality of life

Type 2 diabetes and its complications can have a significant impact on patients' quality of life. The disease can impair patients' life at a cognitive, psychological, physical and social level. The presence of diabetes related acute and chronic complications along with frequent co-morbidities can take a serious toll on patients' well-being (121). Patients with type 2 diabetes frequently have obesity, hypertension, dyslipidemia, cardiovascular disease, renal disease, peripheral vascular disease, neuropathy, retinopathy, sexual dysfunction, all of which can impair their quality of life. Depression can directly contribute to deteriorate patients' quality of life at all levels (121).

The impact of diabetes on the quality of life of patients from culturally diverse populations has been studied. Diabetes and its complications also reduce quality of life in these groups (122, 123). Since co-morbidities and complications are more common among high-risk populations, the impact on their quality of life may even be greater (122, 123). On the other hand and as previously stated, some of these groups are truly family oriented. It has been shown that family support can contribute to improving quality of life in African-American patients with type 2 diabetes (124). Family members should be involved in patient care by providing support when appropriate.

Proper assessment of quality of life requires comprehensive evaluations that are not always feasible in routine clinical care. However, clinicians should get a sense of how type 2 diabetes, co-morbidities and their complications have impacted their patients' quality of life at a physical, psychological, cognitive and social level.

### Religion and faith

Religion and faith are deeply involved in our daily lives and influence our health beliefs and health care behaviors. Faith can be expressed in many ways. Our relationship with an ultimate reality can shape our thoughts, rituals, relationship with others and our participation in society (125).

Religion and faith may influence diabetes care. Sometimes, patients may feel guilty for having developed diabetes. Some may believe the disease is a punishment for what they have done in their lives. Working with patients on accepting the disease and understanding the nature of this condition is important. Improved quality of life has been associated with patients' understanding of the disease process and a forgiving attitude toward themselves (126).

In diabetes care, a clear example of the importance of addressing religious practices pertains to the management of the disease during Ramadan, a time when Muslims fast during the daylight hours for one month each year. Since patients don't drink water and eat during the day, dehydration and hypoglycemia can occur, particularly if they are taking certain glucose-lowering medications. Guidelines on the management of diabetes during Ramadan have been recently published by international organizations (127).

Discussing religion and faith may not be natural for some clinicians. However, it is important to understand patient health-related behaviors that derive from religious practices. Asking patients if they practice any religion and whether it influences diabetes related self-care behaviors should be routinely done in clinical practice.

### Socio-economic status

Poverty clearly influences type 2 diabetes and its complications (128). Socio-economic status is directly linked to the prevalence of type 2 diabetes and hypertension and accounts for as much as 80% of patient related outcomes (129).

Low socio-economic status is associated with higher levels of HbA1c, diastolic blood pressure and LDL-cholesterol, all of which are clearly linked to increased risk for vascular complications (130). High amputation rates in racial/ethnic minorities with type 2 diabetes are closely linked to family poverty (131). Furthermore, socio-economic status was found to be related to the development of cardiovascular disease assessed through coronary calcification in non-Hispanic whites, non-Hispanic blacks, Hispanics and Chinese who took part of the Multi-Ethnic Study of Atherosclerosis (132). Multiple metabolic and vascular disorders have been associated with socio-economic and acculturation factors contributing to the compelling evidence of social determinants on health and disease (133, 134).

Clinicians should consider patients' socio-economic status in the development of type 2 diabetes prevention and treatment programs. Being mindful of patients' financial challenges is recommended in routine diabetes care. The cost of implementing nutritional and exercise recommendations, glucose monitoring practices and the use of medications must be discussed with patients from a financial standpoint.

Addressing social determinants of disease has paramount importance to favorable impact diabetes care in the general population. Targeted strategies in high-risk groups are required.

### Technology

Diabetes information technology programs have contributed to improve diabetes self-care behaviors. For instance, among obese patients with type 2 diabetes, a web-based patient education program led to an improvement in nutrition patterns and some patient related outcomes (135). However, patients with type 2 diabetes don't routinely participate in e-health activities. Their adoption depends on many patient variables as well as characteristics of the health care system and community where people live. Not surprisingly, minority patients are less likely to participate in these activities (136). Nonetheless, when fully embraced, web-based programs can be more effective than those using printed materials in improving diabetes knowledge in patients with type 2 diabetes (137).

Community health workers have been found to contribute to improve diabetes-related outcomes in patients with type 2 diabetes. A study in Latino/Hispanic and African-American low-income patients with type 2 diabetes led by community health workers showed that a tailored, interactive, web-based, tablet computer-delivered tool was equally effective as printed materials in improving knowledge about anti-hyperglycemic medications (138). Interestingly, patients using e-health tools reported higher satisfaction with medication information and lower diabetes related distress than the group exposed to printed materials (138). Thus, incorporating community health workers and/or peers into web-based projects may help in reducing barriers with the use of technology among patients with type 2 diabetes and improve their participation in education and clinical activities.

Successful e-health programs should go beyond proper technology. They must consider frequent communication, bidirectional feedback, and multimodal delivery of the intervention. In addition, they must address the unique socio-cultural-linguistic characteristics among racial/ethnic minorities (139). It would be ideal to consider all factors discussed in this article when developing technology programs for the management of type 2 diabetes in culturally diverse populations.

### Unconscious bias

Unconscious bias, often referred as implicit bias is a prejudice or unsupported judgment against or in favor of a thing, individual, or group (2). People are not usually aware of biases and are not easily controlled.

Biases against racial/ethnic minorities are common in society and in the health care system. Clinicians often exhibit biases that are believed to contribute to health care disparities (12, 140). The same levels of implicit bias against Black, Hispanic/Latino and dark-skinned people in the wider population are present among health care providers (140, 141). Although associations between implicit bias and health care outcomes are not always found, its presence usually affects the interaction between the health care provider and the patient. Unconscious bias may also influence treatment decisions and adherence to therapy (140). Implicit bias is a complex condition that is difficult to identify and eradicate (141).

Medical school, residency and fellowship programs are more frequently addressing the issue of unconscious bias in health care. Continuing medical education programs must also discuss this important factor among health care providers to improve patient-provider interaction. Clinicians must make efforts to identify implicit biases in their clinical practice. Strategies to reduce the influence of biases in their interactions with patients must follow (142). These interventions are required to eliminate health care disparities in the US and other countries around the globe.

### Vulnerable groups

Whereas this work has focused on racial/ethnic minorities within the framework of culturally diverse populations, it is important to remember that many other groups affected by diabetes can be considered vulnerable groups as well. Regardless of their race/ethnicity, the elderly, children/adolescents, LGBTQ groups, pregnant patients, patients with low socio-economic levels, patients with low health literacy/education, and patients with low adherence to therapy and/or depression/emotional distress face many unique challenges in managing their disease. Clinicians should seek resources and recommendations to manage type 2 diabetes in these other special populations.

### Why?

Many patients have their own explanatory model of illness. It is important for all health care providers to ask patients why they think a symptom, a disease and/or a health-related situation may be happening. For instance, rather than telling patients why their blood glucose levels are elevated, it is recommended to ask them why they think their diabetes is not well controlled. Sometimes, they can clearly identify factors that have increased their blood glucose levels. Other times, they may not be certain or may have a wrong idea. They may consider that a medication, stress and/or anxiety are the culprit. Whatever the situation may be, identifying what patients have in their minds is beneficial to effectively gauge conversations and involve them in their health care. Therefore, asking patients why they think something is happening is always a reasonable approach.

Exploring patients' interpretation of the presence and course of their illness, their social environment, their fears, goals and taking their opinion into consideration when designing treatment programs is likely to improve health care behaviors (143, 144).

### Exercise

Physical activity can help delay, prevent, and manage type 2 diabetes. Unfortunately, a small percentage of the population is engaged in regular physical activity. One in four adults between the age of 50 and 64 years and one in three above the age of 75 years reported no physical activity in the prior month in a study conducted by the Centers for Disease Control in the US (145). The lack of physical activity is 1.5 times higher among Latinos/Hispanics and non-Hispanic blacks than in non-Hispanic whites (145, 146). Physical activity increases in parallel to educational attainment (145).

Physical activity is influenced by race/ethnicity and cultural background (147). Some Latinos/Hispanics may not easily engage in jogging or gym exercises while dancing may be preferred (3, 148–151). Some African-Americans patients may like playing basketball or baseball. Physical activity type, frequency and intensity should be routinely discussed with patients and their preferences should be taken into consideration when integrating an exercise program for them. Other factors must be considered as well such as glycemic control, the presence of co-morbidities and complications such as cardiovascular disease, peripheral vascular disease, neuropathy and eye disease, among others (152).

Overcoming barriers to physical activity is extremely important to improve the health status of the population. Identifying community-based programs that help patients engage in regular physical activity is required. Communities need support to develop the required infrastructure to promote and engage patients and families in regular physical activity. Providing patients with specific options of how to engage in physical activity in their own community is crucial.

### “You are in charge”

Sometimes, patients may think their health is entirely their health care providers' responsibility. We as health care providers think patients should have the predominant role and must genuinely engage in adequate self-care behaviors. Employing strategies to empower patients and make them feel they are really in charge of their disease is an important approach to diabetes care. Personal and cultural factors influence the perception of roles and responsibilities for both patients and providers. An open discussion with patients about roles and expectations is recommended as early as possible. This conversation should always take place in a culturally appropriate manner.

The ultimate goal for patients and providers is to engage in an effective shared decision-making process. Clinicians' recommendations must be evidence-based but patient values, preferences and cultural and social context are equally important to develop treatment plans for patients with type 2 diabetes from culturally diverse populations (153).

### Zip it!

Respectfully, I have to say that sometimes we speak too much during clinical encounters and don't give enough time to patients to express their feelings and thoughts. The basis for a trustworthy relationship with our patients is to create the proper setting to listen to each other.

Clinicians usually select a patient problem to explore before allowing patients to express their full concerns. According to a study among family physicians in the United States and Canada, patients are asked to express their concerns in three out of four clinical encounters (154). However, these clinicians interrupted patients in their opening statement after a mean of 23.1 s! Interestingly, patients who were given the opportunity to express their full concerns used only 6 s more on average than those who were interrupted. Those patients who were not asked to express their concerns during the clinical encounter or were interrupted usually had to raise their concerns at a later stage, frequently using more time than if they had been allowed to do so at the beginning. Asking patients about their concerns and allowing them to fully express them adds very little time to the clinical encounter and can improve patient-provider communication (154).

It is difficult to allow patients to fully express their thoughts and feelings in clinical encounters that are often brief and as the pressure to see more and more patients increases in the health care system. However, dedicating some of the time we have with patients to get to know them better will always pay off. Fortunately, health care professionals are slowly recognizing the importance of transitioning from a conventional hierarchic approach to one in which the patient is truly listened to (155).

## Conclusions/summary

Health care providers around the world currently face the challenge of providing care to many patients with type 2 diabetes from culturally diverse populations. Clinicians and their patients must efficiently select the problems and contributing factors to be discussed and addressed in each clinical encounter.

The enormous challenge of eliminating health care disparities requires the development of effective skills and strategies to interact with each other respecting our differences. We must do so in the context of health care systems that don't sufficiently embrace the provision of culturally oriented care yet.

It is imperative to identify and understand our patients' unique biological, psychological, social and cultural characteristics. If successful, we will be able to design comprehensive diabetes prevention and treatment programs in a collaborative and effective manner.

## Author contributions

The author confirms being the sole contributor of this work and approved it for publication.

### Conflict of interest statement

The author declares that the research was conducted in the absence of any commercial or financial relationships that could be construed as a potential conflict of interest. The reviewer JB and handling Editor declared their shared affiliation.

## Footnotes

^1^www.idf.org. Ethnic specific values for waist circumference (Accessed Feb 2, 2018).
